# Mental Health in Australia: Psychological Distress Reported in Six Consecutive Cross-Sectional National Surveys From 2001 to 2018

**DOI:** 10.3389/fpsyt.2022.815904

**Published:** 2022-04-01

**Authors:** Joanne Enticott, Shrinkhala Dawadi, Frances Shawyer, Brett Inder, Ellie Fossey, Helena Teede, Sebastian Rosenberg, Ingrid Ozols AM, Graham Meadows

**Affiliations:** ^1^Southern Synergy, Department of Psychiatry, Monash University, Melbourne, VIC, Australia; ^2^Monash Centre for Health Research and Implementation, Monash University, Clayton, VIC, Australia; ^3^Monash Business School, Monash University, Melbourne, VIC, Australia; ^4^Department of Occupational Therapy, Monash University Peninsula Campus, Melbourne, VIC, Australia; ^5^School of Primary and Allied Health Care, Monash University, Victoria, VIC, Australia; ^6^Brain and Mind Centre, Sydney Medical School, University of Sydney, Sydney, NSW, Australia; ^7^Mental Health at Work, Melbourne, VIC, Australia; ^8^Centre for Mental Health, School of Population and Global Health, University of Melbourne, Melbourne, VIC, Australia; ^9^Monash Health, Dandenong, VIC, Australia

**Keywords:** psychological distress, mental health services, prevalence, population measures, mental health

## Abstract

**Purpose:**

To examine Australian psychological distress trends from 2001 to 2017/18, including analysis by age, sex, location, and household income.

**Methods:**

Secondary analysis of the working age population (18–64 years) in six successive representative national health surveys. Measures were prevalence of psychological distress at very-high symptom level (defined by a Kessler Psychological Distress Scale (K10) score of 30 or more) and combined high/very-high level (K10 score of 22 or more). Very-high K10 scores are associated with mental health problems meeting diagnostic thresholds in past year.

**Results:**

From 2001 to 2017/18 Australian rates of K10 very-high distress rose significantly from 3.8 to 5.1% and combined high/very-high from 13.2 to 14.8%. In women aged 55–64, very-high distress rose significantly and substantially from 3.5 to 7.2% and high/very-high distress from 12.4 to 18.7%. In men aged 25–34, very-high distress increased from 2.1 to 4.0% and high/very-high from 10.6 to 11.5%. Income was strongly and inversely associated with distress (lowest vs. highest quintile adjusted OR 11.4). An apparent association of increased distress with regional location disappeared with adjustment for income.

**Conclusion:**

Australia’s population level of psychological distress increased significantly from 2001–2017/18, with levels highest in women and with rates inversely associated with income. This is likely to be indicative of increased community rates of mental disorders. Given that this has occurred whilst mental healthcare expenditure has increased, there is an urgent need to reconsider how best to respond to mental illness, including targeting the most vulnerable based on social determinants such as age, gender, and lower incomes.

## Introduction

### Monitoring Australia’s Mental Health

Australia has had two instances of a National Survey of Mental Health and Wellbeing (NSMHWB); one in 1997 (*n* = 10,641, response rate 78%) and another in 2007 (*n* = 8,841, response rate 60%) (1, 2) while a further survey with some similarities is underway (3). These provide valuable in-depth cross-sectional information including administration of lengthy symptom-based interviews designed to elicit Diagnostic and Statistical Manual of Mental Disorders (DSM) and International Classification of Diseases (ICD) diagnoses. However, the relative infrequency and irregularity of these surveys, changes in instrumentation, and variability in response rates mean that, for valuable surveillance information on trends in psychological distress and mental disorders in Australia, we need to look elsewhere.

The Australian National Health Survey (ANHS) is an important source of data on health and social determinants (4) which through this century has usually been conducted every 3 years. Typically ANHS sample sizes exceed 20,000 with response rates around 80%. The ANHS includes the Kessler-10 (K10) questionnaire (5). The K10, commonly described as measuring psychological distress, is a ten-item Likert scale, items having a timeframe of 4 weeks and asking how often symptoms occurred in that time. Scoring points range from “all of the time” (5) through “most of the time” (4), “some of the time” (3), “a little of the time” (2), and “none of the time” (1). Total scores range from 10 to 50. Buoyed by the World Health Organization’s World Mental Health Survey Initiative that began in the early 2000s, there is K10 stratum data from over 40 countries, which has enabled estimations of population mental health and comparisons (5–11).

The construction of psychological distress as measured by the K10 includes symptoms commonly associated with common mental disorders, particularly when these are endorsed at higher frequency levels. There are content similarities with symptoms in diagnostic criteria for anxiety and affective disorders but also with responses to other disorder states. Examples here would be K10 items 2, 3, 7 and 9 with specific wordings being of feeling: “nervous”; so nervous that nothing could calm you down”; “depressed”; and “so sad that nothing could cheer you up.” The timeframe of 4 weeks prior where items are scored 4 or 5 involves persistence of symptoms for longer than required to meet diagnostic criteria for an episode of depression (12, 13). Therefore, it appears reasonable that high scores on the K10 would correlate with active common mental disorders. In fact, elevated K10 scores correlating with common mental disorders are reported from the 2007 NSMHWB across Australia (5, 7). In this survey, 79.6% of people with a K10 score in the very-high distress range (scores of 30 and above) had a 12-month CIDI assessed mental disorder (Positive Predictive Value or PPV) and the Stratum-Specific Likelihood ratio (SSLR: probability of a person who has the disease testing positive divided by the probability of a person who does not have the disease testing positive) for any mental disorder was 15.6. High K10 scores (scores of 22–29) had a lower PPV for any mental disorder of 57.1%, with a lower SSLR of 5.3 (7). Therefore, ANHS-based population rates of very-high K10 score represent a reasonable regular survey proxy for recently active mental health problems and our best available measure of this collected regularly in representative surveys in Australia. Combined high/very-high scores provide a measure more broadly of psychological distress rates. This regular ANHS collection of K10 data is currently Australia’s best source for surveillance of mental disorder trends along with those of a broader construct of psychological distress in the Australian population.

#### Previous Work on Time Trends

Previous published work examined trends in psychological distress as measured by the K10 in the adult Australian population from the ANHS between 2001 and 2014 and reported stable rates (14). Headline ANHS rates of very-high K10 as reported by the Australian Bureau of Statistics until 2017/18, so with a further survey data point than in previous reporting, do seem to have increased (up from 2014/15 by 1.3%: from 11.7 to 13.0% for combined high/very-high K10 scores) (15); however comparisons of rates were not standardized for demographic changes. So time trends found in simple rate comparisons could reflect altered population structure rather than valid secular trend findings. Examination focusing primarily on a large Australian nationally representative household panel study with a focus on workforce issues (16) - and with a timespan extending to 2017/18 - did indicate an increase in elevated K10 scores, also commenting on some increase in the ANHS findings for elevated K10. But these comparisons did not apply standardization to the ANHS data for demographic changes.

#### International Comparisons

In a review of major surveys conducted in Australia, Canada, the United Kingdom, and the United States, and in the context of appreciable funding increases for mental health services during recent times particularly in Australia (17, 18), again no improvements in population health were observed (19). We note a possible different picture across some of Europe, as recent analysis of the European Social Health Survey show that in most countries of Europe between 2006 and 2014 the population rates of symptoms associated with depressive disorders seem to have declined (20). The recent comprehensive review of the national burden of 12 mental disorders in 204 countries has examined up-to-date information on the prevalence and burden of mental disorders across the world between 1990 and 2019. No marked changes were found in age-standardized prevalence of any mental disorder (including anxiety and depressive disorders) in any country between 1990 and 2019 (21). However, a limitation of its Australian finding applies as the most recent input data meeting the inclusion criteria (of providing mental disorder prevalence from probability sampling to capture a representative sample of the general population) was obtained in 2007.

### Timing of This Work

The COVID-19 pandemic represents an adventitious event without parallel during the period of history of modern survey methodologies in mental health. Considerable volumes of work have gone on in the context of this pandemic to assess its impact on aspects of mental health; this is critically important, and also important is to understand the trends underway in the mental health of a nation before the pandemic took hold and create an evidence baseline for ongoing population mental health surveillance.

During the previous two decades before the COVID-19 pandemic, Federal and state governments in Australia had increased constant-dollar per-capita mental health services expenditure by 50% (22). Reducing population rates of mental illness featured as an aspiration in key Australian Federal and State policy mental health policy documents [e.g., (23, 24)]. Reducing psychological distress in the population as measured with the K10 was also documented as an intention for the State of Victoria’s 10-year Mental Health Plan (24). Therefore, it is important to report K10 band score population rates regularly. By this, the trends hoped for in policy may be identified and acknowledged if policy implementation is successful, while there can be holding of governments to account if progress is not achieved.

Given that mental health services were accessed by an estimated 12% of Australian adults prior to the pandemic (25), there is appreciable opportunity for treatment services to influence the course of mental health problems and impact population mental health outcomes in Australia. Treatment services may not prevent case onsets, but where a mental disorder within the last year has been detected and effectively treated, we might expect that K10 scores will reduce over time from the higher ranges more rapidly than they would have done without this treatment. Inadequate treatment of an established disorder may be associated with persistent symptoms apparent as elevated K10 scores. Therefore, improved case ascertainment and treatment might reasonably be expected to reduce surveyed rates of very-high K10 scores. If effective treatment rates increase, then more people with the identified problems will have, with support by treatment and care, transitioned from the higher to lower rates of symptomatology reflected in K10 scores. Noting here that the K10 is one of the instruments advised for use as an outcome measure in Australian primary mental health care, (26) we might hope to find population mental health improvement in the previous two decades when funding for treatment services increased substantially. Given that further increases in mental health services spending are now occurring as part of the response to the mental health impacts arising from the COVID-19 pandemic, it is critical to explore and understand what impact previous increases in mental health expenditure had on population measures if any. This can help to inform future services spending and support the implementation of evidence based initiatives to support improvements population mental health.

### Regular Population Mental Health Surveillance in Australia and the Aims of This Work

Previous work has reported overall ANHS rates of K10 score bands up to 2014 (14, 27) – this work adds by inclusion of a further national survey data point and, like that reporting, applies standardization for population changes. Also, adding to previous work (14, 28, 29), we examine prevalence of psychological distress in Australia between 2001 and 2018, exploring subgroups by age, gender, household income and location. In order for the relationships with income to be coherently examined and consistent with other data presented we restrict analyses to the working age population.

## Materials and Methods

### Design

This study was a large-scale secondary analysis (*n* = 78, 204) of K10 data collected by the Australian Bureau of Statistics (ABS) from working-age Australian adults across six National Health Surveys (ANHS) (2001-02, 2004-05, 2007-08, 2011-12, 2014-15, and 2017-18). We analyzed responses from adults aged 18–64 years in each survey, except for the 2004-05 ANHS as data was only available for adults aged 20–64 years. We standardized all surveys to the 2001 Australian census population based on the strata of sex and age (30). Elevated psychological distress rates were calculated and compared across sex as available in the ANHS.

### National Health Surveys

These ANHS cross-sectional household-based surveys are undertaken at 3-year intervals to monitor health trends over time with detailed methods described elsewhere (4). Trained ABS interviewers conducted face-to-face interviews in each survey. Household and person weights are assigned by the ABS to adjust for the probability of sample selection, seasonality and non-response, and the data are then calibrated to the population benchmarks. This ensures that the estimates are representative of population distributions and compensates for any over- or under-representation of particular categories of persons or households.

### Psychological Distress Measure

The K10, a self-administered 10-item Likert scale tool, measures current psychological distress, particularly symptoms of anxiety and depressive disorders (5). Used in ordinal form, band scores are closely associated with mental health disorders (5). K10 scores range between 10 and 50, and score bands are: low (10–15), moderate (16–21), high (22–29), and very-high (30–50). Here we also generated an overlapping and combined high/very-high category, which consisted of scores 22 and higher.

### Geographic Location

A residential location variable for each survey participant is available and based on the Accessibility and Remoteness Index of Australia (ARIA+) (4). It describes the residential location as Major cities of Australia, Inner Regional Australia or Other.

### Data Analysis

All statistical analyses were performed in Stata 16.0 (StataCorp, College Station, TX, United States). When not stratified by age, data were directly age-standardized against the estimated resident population of Australia as at 30 June 2001. Using this direct age-adjustment approach, the 2001 age-structured population is used as the reference and each survey round is weighted to match this (30). Effect size estimates for dichotomous outcomes of combined high/very-high and very-high psychological distress are presented as odds ratios calculated using logistic regression on the K10 data from the Australian working age population. Independent variables examined first in a univariate regression with the outcome, then in a multivariable regression, were: year, sex, age-group, household income, and location. All independent variables were specified as categorical, including the “year” variable because prevalence changes over time was not linear. For time trend examinations the reference year was 2001. The overall time trend examinations done using the regression analyses had a level of significance set at an alpha of 0.05. Subsequent sub-group pairwise comparisons using 2001 and 2017-18 data employed tests for two proportions. Given that twelve sub-group pairwise comparisons were planned (see the section “Results”), to minimize the occurrences of spurious positives a Bonferroni correction was applied with the alpha value was set at 0.0042 (i.e., approx. 0.05/12).

### Ethics Approval

As is common practice for the ABS, data collection occurred under the auspices of the Census and Statistics Act 1905. Per the ABS and Universities Australia Agreement (31), students, staff, and researchers affiliated with participating universities have access to the basic, anonymized, microdata for the 2001-02, 2004-05, 2007-08, 2011-12, 2014-15, and 2017-18 cycles of the ANHS. Therefore, ethics approval was not required for these analyses.

## Results

### Overall Results and Time Trends

**FIGURE 1:**
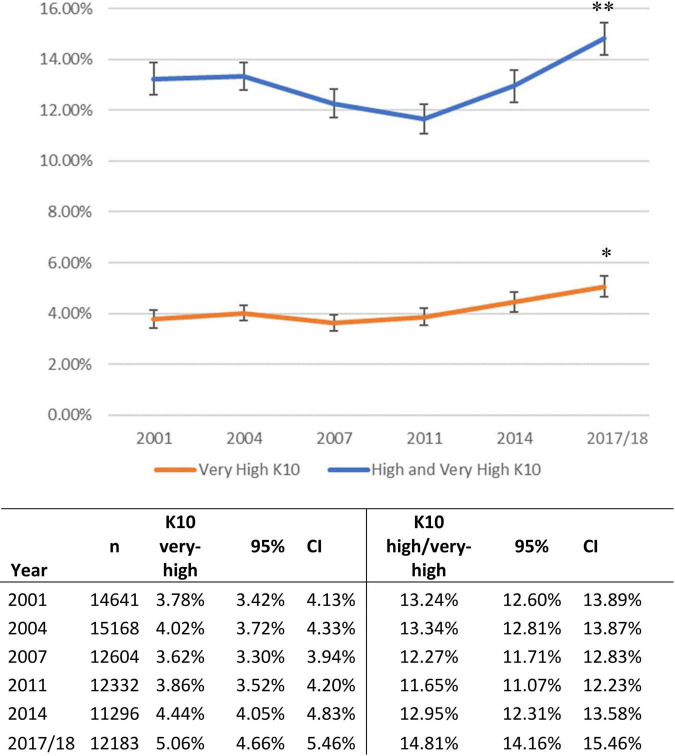
Age-standardized prevalence of psychological distress in the Australian working age population, 2001–2017. ^a^Standardized to 2001 Australian Census. Derived from a total of *n* = 78,204 survey participants aged 18–64 years. ^**^Rate at 2017 significantly greater than all previous years (*p* < 0.001). * Rate in 2017 significantly greater than 2001, 2004, 2007, and 2011 (*p* < 0.01).

In the six national surveys between 2001 and 2017-18 there were *n* = 78,204 surveys completed by working-age adults producing K10 distress data, see Table 1. Figure 1 shows that the greatest distress occurred in the latest survey at 2017-18: for combined high/very-high level distress the 14.8% rate was significantly greater than all previous years (*p* < 0.001); for very-high level distress the 5.1% rate was significantly greater than 2001, 2004, 2007, and 2011 (*p* < 0.01).

**TABLE 1:** Age-standardized prevalence of psychological distress in the Australian working age population, 2001–2017/18.

	K10 very-high				K10 combined high/very-high		
		
	n^s^	Rate^a^	95%	CI	Rate^a^	95%	CI
***Age Group***							
18 – 24	7846	4.05%	3.62%	4.49%	15.75%	14.95%	16.56%
25 – 34	17292	3.55%	3.27%	3.82%	12.78%	12.28%	13.28%
35 – 44	19874	3.97%	3.69%	4.24%	12.99%	12.52%	13.46%
45 – 54	17742	5.39%	5.05%	5.72%	14.00%	13.49%	14.51%
55 – 64	15470	4.53%	4.20%	4.85%	12.96%	12.43%	13.49%
**Location*^a^***							
Major cities	51289	4.09%	3.92%	4.27%	13.13%	12.83%	13.43%
Inner regional	15073	4.75%	4.40%	5.10%	14.42%	13.83%	15.00%
Other	11862	4.24%	3.87%	4.61%	13.66%	13.01%	14.30%
***Sexr^a^***							
Male	36809	3.30%	3.12%	3.49%	10.90%	10.57%	11.22%
Female	41415	5.18%	4.96%	5.41%	16.05%	15.68%	16.42%
***Male and Year*^a^****							
2001	6797	3.01%	2.60%	3.41%	10.86%	10.11%	11.61%
2004	7135	3.27%	2.86%	3.69%	11.33%	10.57%	12.08%
2007	6095	2.87%	2.46%	3.28%	10.29%	9.52%	11.06%
2011	5871	3.08%	2.63%	3.52%	9.54%	8.77%	10.31%
2014	5197	3.43%	2.92%	3.93%	10.35%	9.50%	11.19%
2017/18	5714	4.19%	3.66%	4.72%	12.57%	11.68%	13.46%
***Female and Year*^a^****							
2001	7844	5.19%	4.69%	5.69%	17.00%	16.15%	17.84%
2004	8033	5.02%	4.54%	5.50%	16.73%	15.89%	17.58%
2007	6509	4.59%	4.08%	5.11%	15.33%	14.44%	16.23%
2011	6461	4.61%	4.09%	5.13%	13.72%	12.85%	14.58%
2014	6099	5.43%	4.85%	6.02%	15.50%	14.55%	16.44%
2017/18	6469	5.90%	5.31%	6.50%	16.97%	16.03%	17.92%
***Household income quintiles***							
(poor) 1	10031	10.88%	9.25%	12.51%	24.56%	22.36%	26.76%
2	10050	6.06%	4.78%	7.35%	20.11%	17.95%	22.26%
3	13307	1.98%	1.41%	2.56%	11.82%	10.27%	13.37%
4	15601	2.14%	1.47%	2.81%	9.12%	7.82%	10.43%
(rich) 5	16897	1.01%	0.58%	1.45%	5.76%	4.82%	6.70%
***Female and Household income quintiles***							
(poor) 1	5915	11.91%	9.78%	14.03%	26.94%	24.06%	29.82%
2	6005	6.80%	5.07%	8.52%	23.31%	20.37%	26.24%
3	7078	2.53%	1.59%	3.46%	14.99%	12.55%	17.43%
4	7840	2.75%	1.66%	3.84%	11.44%	9.38%	13.49%
(rich) 5	7736	1.75%	0.92%	2.58%	7.79%	6.16%	9.42%
***Male and Household income quintiles***							
(poor) 1	4120	9.64%	7.13%	12.15%	21.72%	18.35%	25.08%
2	4050	5.12%	3.22%	7.03%	15.99%	12.86%	19.11%
3	6229	1.45%	0.77%	2.13%	8.70%	6.83%	10.58%
4	7761	1.54%	0.75%	2.33%	6.88%	5.29%	8.47%
(rich) 5	9161	0.43%	0.01%	0.85%	4.16%	3.09%	5.24%

*^a^Standardized to 2001 Australian Census. Derived from a total of n = 78,204 survey participants aged 18–64 years. ^S^Number of survey respondents.*

For very-high distress, multivariable regression identified similar rates of very-high distress across 2001, 2004, and 2007, see Table 2. Then compared to 2001, greater rates were evident in 2011, 2014 and 2017-18 with odds ratios (OR) of 1.15 (95% CI: 1.001–1.33), 1.21 (1.06–1.39) and 1.40 (1.23–1.59), respectively.

**TABLE 2:** Odds ratio of psychological distress adjusted for year, sex, age, residential location, and household income.

	K10 very-high					K10 combined high/very-high			
		
		Odds Ratio	*p*-value	[95% Conf.	Interval]	Odds Ratio	*p*-value	[95% Conf.	Interval]
Year	2001	(Ref)	–	–		(Ref)	–	–	
	2004	1.06	0.366	0.93	1.20	1.05	0.165	0.98	1.13
	2007	1.01	0.871	0.88	1.16	0.99	0.723	0.91	1.07
	2011	1.15	0.048*	1.00	1.32	0.91	0.025*	0.84	0.99
	2014	1.21	0.006**	1.06	1.39	1.00	0.998	0.92	1.09
	2017/18	1.40	<0.001***	1.23	1.59	1.18	<0.001***	1.09	1.27
Sex	Males	(Ref)	–	–		(Ref)	–	–	
	Females	1.39	<0.001***	1.28	1.50	1.39	<0.001***	1.33	1.46
Age	18 – 24	(Ref)	–	–		(Ref)	–	–	
(years)	25 – 34	0.97	0.712	0.83	1.14	0.86	0.001**	0.79	0.94
	35 – 44	1.06	0.440	0.91	1.24	0.86	0.001**	0.79	0.94
	45 – 54	1.48	<0.001***	1.27	1.72	0.97	0.517	0.89	1.06
	55 – 64	0.95	0.550	0.82	1.11	0.72	<0.001***	0.66	0.79
Location	Major cities	(Ref)	–	–		(Ref)	–	–	
	Inner regional	0.98	0.733	0.89	1.08	0.93	0.024*	0.88	0.99
	Other	0.86	0.007**	0.77	0.96	0.90	0.001**	0.84	0.96
Household income quintile	Richest	(Ref)	–	–		(Ref)	–	–	
	4	1.52	<0.001***	1.27	1.81	1.52	<0.001***	1.39	1.65
	3	2.47	<0.001***	2.09	2.92	2.03	<0.001***	1.87	2.20
	2	5.54	<0.001***	4.73	6.48	3.53	<0.001***	3.25	3.82
	Poorest	11.54	<0.001***	9.94	13.39	6.22	<0.001***	5.76	6.72

***<0.001, **<0.01, and *<0.05. Income quintile 1 are lowest incomes, and quintile 5 are highest.

For combined high/very-high distress, multivariable regression identified that compared to 2001, the 2011 rate was significantly lower with OR of 0.91 (0.84–0.99), whilst in 2017-18 rate was greater with OR of 1.18 (1.09–1.27), see Table 2.

### Age and Gender

**FIGURE 2:**
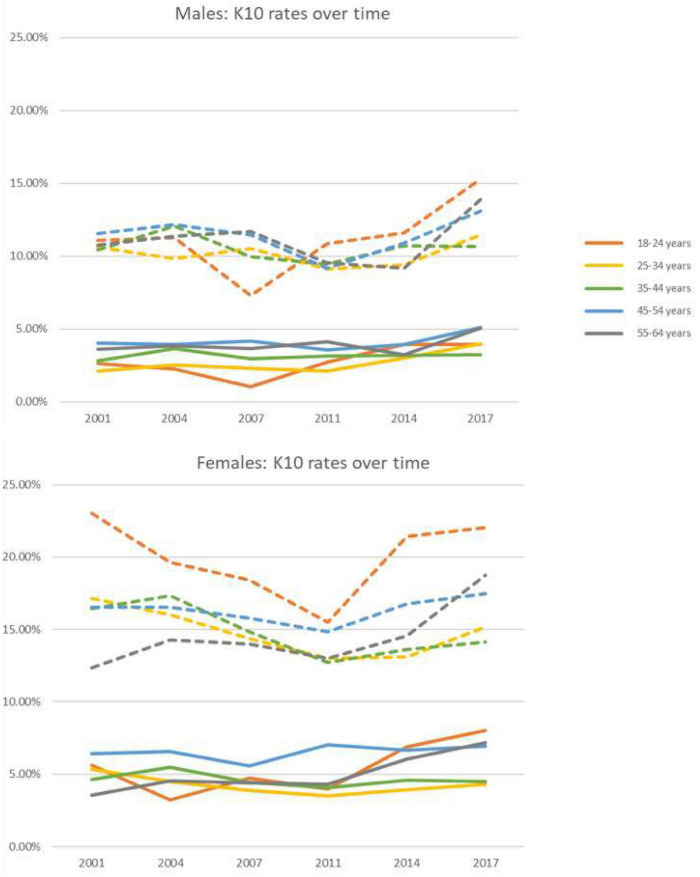
By age-groups and sex, shown are the age-standardized prevalence of psychological distress (solid lines are the Very-high K10; and broken lines are the combined High/very-high K10). Standardized to 2001 Australian Census. Derived from a total of *n* = 78,204 survey participants aged 18–64 years. The 95% confidence intervals are given in Supplementary Table 2.

Figure 2 shows the K10 distress data broken down by age and gender over time. In analysis by gender, very-high distress was more prevalent in women at 5.2% (95% CI: 5.0–5.4) compared to men at 3.3% (95% CI: 3.1–3.5), see Table 1. Combined high/very-high distress was also more prevalent in women at 16.1% (95% CI: 15.7–16.4) compared to men at 10.9% (95% CI: 10.6–13.9). Multivariable regression confirmed that women had greater odds for very-high distress (OR 1.39, 95% CI: 1.28 to 1.50) and for combined high/very-high distress (OR 1.39, 95% CI: 1.33 to 1.46), as compared to men, see Table 2.

In analysis by age groups, very-high distress rates ranged between 3.6% (95% CI: 3.3–3.8) in those aged 25–34 years to 5.4% (95% CI: 5.1–5.7) in those aged 45–54 years, see Table 1. Multivariable regression showed that only the 45–54 age group [5.4% (95% CI: 5.1–5.7)] had significantly greater odds for very-high distress (OR 1.48, 95% CI: 1.27 to 1.72) compared to the youngest group at 4.1% (95% CI: 3.6–4.5), see Table 2. Prevalence of combined high/very-high distress was greatest in those aged 18–24 years at 15.8% (95% CI: 15.0–16.6). Multivariable regression confirmed that most other age-groups had significantly lower rates than those aged 18–24 years with an OR of 0.86 (95% CI: 0.79–0.94), 0.86 (95% CI: 0.79–0.94), and 0.72 (95% CI: 0.66–0.79) for 25–34, 35–44, and 55–64 years, respectively, see Table 2.

The results of the pre-specified two-sample comparisons of the first and the last surveys (2001 and 2017/19) are shown in Table 3. Positive differences indicate an increase in prevalence in 2017/18 compared to 2001. Given that twelve sub-group comparisons were planned, to minimize the occurrences of spurious positives, the alpha value was set at 0.0042. The most marked increase in psychological distress between 2001 and 2017/18 is seen in women aged 55–64 years old, with very-high distress in 2001 at 3.5% (95% CI: 2.5–4.56) up to 7.2% (95% CI: 5.9–8.5%) in 2017 (Figure 2 and Supplementary Table 2). This doubling of prevalence was highly significant with a difference of 3.7% (*z* = 4.10, *p* < 0.0001). Combined high/very-high distress also significantly increased from 12.4% (95% CI: 10.5–14.2%) in 2001 to 18.7 (95% CI: 16.7–20.7%) in 2017. This increase of prevalence was highly significant with a difference of 6.4% (*z* = 4.51, *p* < 0.0001). Another almost doubling of combined high/very-high distress between 2001 and 2017 is seen in men aged 25–34 years old, with very-high distress in 2001 at 2.1% (95% CI: 1.4–2.8) up to 4.0% (95% CI: 2.9–5.1%) in 2017, which was also significant with a difference of 1.9% (*z* = 2.87, *p* = 0.002).

**TABLE 3:** Two-sample comparisons of psychological distress rates between the first and the last surveys in 2001 and 2017/19.

	K10 very-high				K10 combined high/very-high			
		
	Difference	*p*-value	95%	CI	Difference	*p*-value	95%	CI
*Male*	1.09	0.001*	0.43	1.75	1.71	0.004*	0.55	2.87
*Female*	0.71	0.067	−0.05	1.47	0.03	0.963	−1.23	1.29
***Male***								
18 – 24	1.27	0.045	0.03	2.51	4.23	0.023	0.57	7.89
25 – 34	1.86	0.003*	0.62	3.10	0.84	0.925	−16.59	18.27
35 – 44	0.40	0.505	−0.77	1.58	0.21	0.957	−26.59	28.27
45 – 54	1.09	0.160	−0.43	2.61	1.15	0.179	−0.72	3.81
55 – 64	1.42	0.090	−0.22	3.06	3.14	0.022	0.449	5.83
***Female***								
18 – 24	2.37	0.252	−1.70	6.44	−0.96	0.655	−5.06	3.14
25 – 34	−1.05	0.149	−2.48	0.379	−1.98	0.133	−4.57	0.61
35 – 44	−0.16	0.826	−1.59	1.27	−2.32	0.047	−4.61	−0.03
45 – 54	0.51	0.995	−183.5	183.4	0.95	0.462	−1.59	3.49
55 – 64	3.63	<0.0001*	1.94	5.32	6.35	<0.0001*	3.45	9.33

Positive differences indicate an increase in prevalence in 2017-18 compared to 2001. Given that twelve sub-group comparisons were planned (see Table 2), to minimize the occurrences of spurious positives, the alpha value was set at 0.0042. * indicates p ≤ 0.0042.

### Income

In terms of household income, very-high distress was significantly more prevalent in those in the poorest quintile at 10.9% (95% CI: 9.3–12.5) compared to all other quintile groups, see Table 1. The richest quintile had the least prevalent rate of combined very-high distress at 1.0% (95% CI: 0.6–1.5). Combined high/very-high distress was also significantly more prevalent in the poorest quintile at 24.6% (95% CI: 22.4–26.8) compared to all other quintiles. The richest quintile had the least prevalent rate of combined high/very-high distress at 5.8% (95% CI: 4.8–6.7). Multivariable regression (Table 2) found those in the poorest household income quintile to have the greatest odds for very-high distress (OR 11.54, 95% CI: 9.94–13.39) compared to the richest quintile; and greatest odds for combined high/very-high distress (OR 6.22, 95% CI: 5.76–6.72) compared to the richest quintile.

### Location

In terms of geographical location, very-high distress was more prevalent in those residing in inner regional areas at 4.8% (95% CI: 4.4–5.1) compared to major cities at 4.1% (95% CI: 3.9–4.3), see Table 1. Combined high/very-high distress was also more prevalent in inner regional areas at 14.4% (95% CI: 13.8–15.0) compared to major cities at 13.1% (95% CI: 12.8–13.4). Multivariable regression (Supplementary Table 1) adjusting by age and sex found those in inner regional areas to have greater odds for very-high distress (OR 1.16, 95% CI: 1.06–1.26) compared to capital cities; and greater odds for combined high/very-high distress (OR 1.11, 95% CI: 1.05–1.17) compared to capital cities. When income is added into the regression (Table 2), however, this association disappears (OR 0.98, 95% CI 0.89–1.08). For combined high/very-distress, the OR then reverses is in favor of lower distress in inner regional (OR 0.93, 05% CI: 0.88–0.99), and other regions (OR 0.90, 95% CI: 0.84–0.96).

## Discussion

### Key Trend Findings

In Australia from 2001 to 2018, levels of very-high psychological distress significantly rose from 3.8% at the start of this period to 5.1% at the end. Combined high/very-high distress increased from 13.2 to 14.8%. A modest rate of decline in distress during the late 2000s was unsustained. After adjusting for age, sex, location and income, very-high distress was significantly more prevalent in 2011, 2014 and 2017/18 as compared to 2001; and high/very-high distress was significantly greater at 2017/18 as compared to 2001.

#### Changes Vary Between Subgroups

Very-high distress in women aged 55–64 has doubled this century (from 3.5 to 7.2%) and combined high/very-high distress has increased by 50% (12.4–18.7%), both of which are highly significant findings. Very-high distress also increased in males, significantly in those aged 25–34 years, but this is a more tentative finding since a significant increase did not extend to the combined high/very high distressmetric (10.6–11.5%). Overall, distress was greatest in women aged 18–24 years during all years; 8.0% for very-high levels and 22.1% for combined high/very-high in 2017/18 (2.1–4.0%). Although this study examined successive cross-sectional national surveys, the individuals in whom the distress has increased would have been younger versions of themselves at the times of the initial comparison survey so the increase in distress should be assessed with this context. At face value, and as much as can be inferred from this data set, these increases do not appear to be due to a birth cohort effect. For women, the 55–64 age group had the lowest prevalence of very-high psychological distress in 2007 (then aged 45–54 years) compared with females of the same age bracket in other years. A decade earlier, the male 25–34 age group (approximated in the 18–24 years age group) also had the lowest prevalence of very-high psychological distress compared with men of the same age bracket in other years. Rather than birth cohort effects, the findings of an increasing rate of psychological distress (and rapidly increasing in women in the 55–64 age subgroup) is concerning and is looking more like arising from adventitious cause(s), which are discussed more further below.

Income was strongly associated with distress, with the largest subgroup prevalence differences seen between the lowest and highest income quintiles. Income is important to examine in analyses of populations as it can be a proxy for many factors, including education, economic environment, and employment. These in turn may also affect access and utilization of mental health services. Without considering income in the analyses, we found significantly greater psychological distress among Australians residing outside of major cities; however this result reverses when income is included. This demonstrates the evident protective effect of higher incomes for mental health, and the fact that people living outside of major cities generally have lower incomes and higher costs than their major city neighbors (32, 33). Across the timespan of the study, distress was very much greater in the context of lower income levels. Indeed, when the effects of population demographics and income are controlled for, a small protective effect of living in inner and outer regional and remote Australia was found.

### Possible Causality and Remedies: Policy Implications

#### Social Policy Implications

The World Health Organization (WHO) has noted that “Mental health and many common mental disorders are shaped to a great extent by the social, economic, and physical environments in which people live” and that social inequalities increase risk of many common disorders (34). Social determinants link with gender, biological and environmental factors, health and other policies to influence incidence of mental health problems, their persistence or otherwise, and related outcomes across the lifespan (34, 35). Possible contemporary negative influences of social determinants on population mental health include: increased job insecurity and casualization of the workforce; financial stress associated with housing affordability (36); increased working hours and disruptions to work life balance; continuing unaddressed intergenerational disadvantage applying to indigenous peoples and other minority and diverse groups; and the pervasive existential threat posed by climate change (34, 37). There is reason to believe that inequalities in society may be associated with worse mental health and wellbeing outcomes across populations for many problems with social gradients (38, 39); cross-nationally, rates of mental illness symptoms are positively associated with income inequality as measured with the Gini index (40). We note that in Australia, income inequity as measured by the Gini index and calculated for weekly income, increased appreciably from 0.304 in 2001-02 to 0.313 in 2017-18 (41). Wealth inequity also increased in Australia between 2003 and 2016, with the most affluent financial quintile experiencing a 53% increase in wealth, and the poorest, a 9% decline (42).

In calling for action to address social determinants of mental health issues, the WHO has argued that action needs to be universal, across the whole of society and proportionate to need, seeking to level the social gradient in health outcomes. Proposed strategies included environmental, structural and local interventions (34). The finding that Australia’s mental health, based on best available national data, has been worsening as the 21st century has unfolded so far, has implications far beyond what is usually regarded as mental health policy. Rather, it should prompt consideration as to changes to wider policy settings across ranges of: taxation, housing, educational, employment, social benefits, and anti-discrimination and reconciliation actions, and even climate policy. There is no simple prescription here, but there is guidance. For example, current fiscal policy has contributed to greatly increasing house prices in Australia and decreasing home-ownership for young people and those with lower incomes (whereas 35 years ago home-ownership rates were high for Australians in all income levels and in younger people too) (36). Those on low incomes – increasingly renters – are experiencing more financial stress by spending more of their income on housing, and intergenerational inequity is being propagated as home ownership for young people is now becoming associated with the wealth of the parents (36). The WHO social determinants framework (34) would suggest that addressing factors such as financial stress and intergenerational inequity could make an important contribution to improving mental health in young adults. Contributing to the finding of increasing psychological distress in women aged 55–64 will be contemporary structural and occupational factors affecting women in this age group such as the impacts of divorce, gender pay gap, carer responsibilities, and insecure work (43–45). Women in this age group are more likely to be at risk of poverty and homelessness in Australia (46), while greater socioeconomic disadvantage of geographical areas where such women may need to live (17, 47) and lower personal income (48) are associated with 2–3 fold increased prevalence of mental health issues. These influences may be contributing to these findings regarding increasing psychological distress in this demographic group (47). Income stress may further compromise access to healthcare services that require co-payments.

Recognition of the fundamental inter-relationship between mental health and the social determinants of health has led several governments both in Australia (49) and elsewhere (50) to develop wellbeing frameworks. These frameworks are designed to use social and environmental indicators, along with economic and fiscal ones, to prioritize mental health and guide Government investment and funding decisions beyond the health system, and into key relevant areas such as employment, housing, education and social inclusion. While proof of the impact of such frameworks is yet to emerge, they demonstrate increased appreciation of the need to promote holistic policy and planning, beyond the confines of the health system.

#### Health Policy Implications

From the perspective of healthcare, advocacy can include broad modifiable societal and social determinants: however, addressing many of these social determinants lies outside the direct influence of healthcare providers, policymakers, or those concerned with institutional care quality. The importance of broader societal changes notwithstanding, given the intent to influence population mental health expressed in national and state policy documents, these findings raise questions about Australian mental health policy and its implementation.

#### Increasing Service Volumes

Service and funding innovations in the first two decades of the 21st century in Australia have led to substantial increases in items of mental health care delivered (28, 51), with an estimated 12% of the population accessing mental health care prior to the start of the pandemic in 2020 (25). In a major expansion of Australia’s national Medicare health insurance scheme from November 2006 onward, the Better Access initiative (28) has enabled a range of non-medical service providers including psychologists, social workers, and occupational therapists, to access rebates through the Medicare scheme. Through the Better Access initiative among others, much of the increased investment in mental health care in Australia has been targeted at care for higher prevalence mental health problems and has led to a very considerable increase in delivery of focused psychological strategies. Success in lifting the rate of access to care for higher prevalence problems is in contrast to state and territory care, principally provided to people with lower prevalence disorders, for which access to care has remained static over the past two decades (52).

#### Mental Health Care Can Be Effective

Contemporary mental health care has a large body of evidence supporting its efficacy and effectiveness. For instance, a range of antidepressant medications can be found to consistently improve outcomes of depression (53), while the same can be said for many forms of psychotherapy in treatment of anxiety, depression, and other common mental health problems (54). Increasing public awareness of mental health and reducing stigma has occurred in Australia (55, 56). So there is a rational causal pathway between scaling up of such interventions to population health delivery and the attainment of positive change in mental health indices in the population. These clinical interventions, if applied, will not necessarily avert new episodes of poor mental health but they can lead to earlier resolution of active symptom status and prevent relapse or recurrence (57–60), which will be reflected in lower overall K-10 psychological distress when measured cross-sectionally in surveys.

So why are things getting worse? It could be argued that recent service changes in Australia might be expected to have had some impact on rates of psychological distress as measured with the K10. Instead, these years (in the 21st century to date before the COVID-19 pandemic) have seen the mental health of Australians worsen appreciably, as measured using psychological distress in national surveys. The likely potent role of changing social determinants in worsening mental health has been discussed above, so now we turn to considering the ways in which the mental health care system may be not functioning well in ameliorating the effect of these determinants, or even possibly contributing to mental ill health.

#### Navigation and Access to Effective Care

Repeated inquiries have found that Australia’s mental health system is hard to navigate (61) and concern has been raised about the likely scale of a quality gap in some mental health service delivery as well as important gaps in access (28). Poor articulation of responsibilities between different levels of government have permitted the evolution of a proliferation of service structures (61, 62). Comprehensive, recovery-oriented and person-centered care is rare (63). Navigation could be assisted by better coordinated services including around collaborative care models (64). The need to develop collaborative approaches to the training of mental health professionals has also been noted as a key to creating the multidisciplinary teams required to respond, particularly to more complex mental health needs (65). Australia is yet to develop such training approaches. There is also a need to promote collaborative mental health research, such as in the evolving field of research and practice that is global mental health (66) which “prioritizes equity, and is informed by many disciplines, including neuroscience, genomics, social sciences (especially psychology, medical anthropology and sociology), epidemiology, health services research, and implementation science.” There have been examples of successful innovations in primary care collaboration in Australia (67) but the division of healthcare responsibility in this country between the federal responsibility for ambulant care through the Medicare insurance system and the state administered hospital and community mental health care systems presents obstacles to making such innovations seamless and sustainable.

#### Targeting Specific Demographics

Specific demographic groups identified here as having rapidly high rates of problems are those aged less than 34 years and females 55–64. There is a longstanding focus in Australia on services for youth mental health and these findings confirm that this phase of life is associated with high levels of mental health problems. We have already introduced some of the social and economic drivers that may be affecting younger people, and it might be speculated that the observed high distress levels could reflect disproportionate impacts of various social factors, such as personal income and relationship stressors, and worsening housing affordability across Australia (36) at a stage of life where young people are often establishing long-term co-habiting relationships and starting families and careers (68–72). This emphasizes that, with services focusing on youth often defined as up to 25, the needs of people who may just miss out on these more intensive services should not be neglected, and furthermore that social, education, employment and mental health programs need better integration to address these needs.

For women aged 55–64, multiple social and economic causes may also converge, given aforementioned increased risks of poverty, homelessness as well as impacts of family violence in this group (44, 47). Therefore, services may need to find ways to better reach out to these women; to integrate practical help around issues, such as homelessness risk and income security, with mental health responses; and to attend to workforce development in areas that may be particularly deficient in response to key influences on mental health in this group of women, including screening for and supporting those experiencing family violence (73).

#### Funding Models and Access

While the Australian health care system is commonly described as universal in nature, the public health insurer Medicare permits providers to charge co-payments, creating manifestly substantial inequities in the delivery of psychological services (51). The Commonwealth Fund recently reported on the health care system from Australia and other high-income OECD countries (74) – the source data was a questionnaire assessment of cost-related access problem to medical care. Australia together with Norway and the Netherlands were the top three overall for health care system performance (74). However, while the gap between higher and lower income groups on a binary split was small, overall the 21% of Australians who identified cost-related access problem to medical care actually ranked third of eleven countries, behind only the United States and Switzerland. Data presented here confirms that those with lower household income have much greater psychological distress, and those on especially low incomes are most likely logically to have income stress associated with their health and mental health care. They also will have other possible barriers to access and participation in care so the approach to evening out these inequities will likely be complex.

Clinical mental health services may well be useful, though only to those who receive them. The challenge for policy and service planning is to encourage access that is proportional and equitable. There is considerable evidence of widespread failure in this regard. Increased care volume in Australia has been demonstrated as misaligned with community needs and not necessarily providing care at consistent quality (17, 18, 28, 33).

#### Inequity, Ineffectiveness, and Iatrogenesis

In turn, these problems may have compromised effectiveness of Australia’s mental health service delivery system quality (28, 29), impacted by inequity in service delivery and forms of iatrogenesis (17, 75). A lack of data precludes the analysis of service quality across much of the service system and this itself is a problem (62). However, greater attention to consistent attainment and assurance of quality care is important going forward. Addressing disparities in mental healthcare outside major cities should be a continuing priority. While the finding from this work was that the increased rate of psychological distress found in regional areas was not retained in analyses when income was controlled for, nevertheless it showed increased distress in the lowest income areas which are often located in regional areas. Service delivery needs to be structured with these needs in mind.

Perhaps inequitable and lower quality treatment for mental health problems may actually do some harm as well as good. It has been suggested that iatrogenic influences based on a loss of agency arising from medicalization might perhaps negate the relatively modest effect of antidepressant monotherapy (17, 75, 76). It also has been proposed that antidepressants themselves may have a significant property of oppositional perturbation, so increasing the rates of depression among those who have been prescribed them above the rates that would have been observed had they never been exposed to this therapy (75). Between them, these two explanations constitute a possible route for understanding why undoubted therapeutic benefits that may flow to some individuals fortunate enough to get access to comprehensive and appropriate care, may be offset by what can be seen as iatrogenic harms for those receiving more limited forms of care, and so constituting a failure of quaternary prevention (77, 78). Is it also worth noting that despite significant new public funding for talk therapies under the Better Access program, the rate of prescribing of antidepressants has increased: it was 11.4/100 persons in 2013-14 and 13/100 in 2019-20 (79).

#### Complexity and New Modeling Approaches

Past planning approaches have failed to reflect the array of influences on population mental health. Newer and more sophisticated approaches are required (80, 81), and a paradigm shift in mental health research is required to achieve further progress (76). Simulation modeling has become a topic of regular household discussion during the pandemic yet is rarely employed in directing mental health planning with the same kind of sophistication with which it has been applied to COVID-19. Such modeling must, and has the capacity to, reflect the range of social determinants we have identified. It can demonstrate how mental health services may be adapted to achieve better outcomes for more people and influence prevalence even in such adverse conditions.

### Limitations

A limitation was our data structure, since age was provided in 5-year bands and the survey was conducted every 3 years, a cohort variable could not be determined. To fully examine cohort effects, an alternative analytic approach would be a full age-period-cohort (APC) analysis. Our analysis plan did follow APC guidance (82), and assumed that the cohort dimension was non-operative (83) based on the above observations of no evidence of birth cohort effects.

We note that further subgroup examination may add to our understanding of the operation of other social determinants, but that is beyond the scope of this paper. We included income and geographical location in our analyses because of the widely known effects between mental health and these variables (48). Although income can be a proxy for many things (education, economic environment, employment and service access, and utilization) further research using these variables as available in the ANHS could expand knowledge in these areas. Additionally, for the ANHS undertaken to date, very remote areas are out of scope. Forthcoming detailed mental health surveys will apply more specifically valid diagnostic instruments (84) but will be smaller and so less able to examine subgroups as here.

Another limitation is the increasing public awareness of mental health and reducing stigma has occurred over this period in Australia (55, 56), which may have contributed to an increased reporting of psychological distress. The argument for use of very-high K10 as a proxy for common mental disorder rates rests partly on content but also on findings from the PPV in the 2007 NSMHW (7). The PPV of the very-high K10 scores may change as the prevalence of ICD-diagnosed mental health disorders in the population changes and the provision by Slade et al. (7) of SSLRS enables us to estimate the possible impact of this. For example, if the prevalence of affective disorders has doubled from Slade et al’s estimation of 6.2% to 12.4%, the PPV of the K10 would increase to an estimated 72%. So an increase in population prevalence of mental disorder would lead to an expected increase in the PPV for very-high K10 rather than a decrease.

We note here that these data sources pre-date the COVID-19 pandemic. The impacts of COVID-19 are significant and mental health impacts including increased psychological distress have been reported to be greater in women (85); however, this will be reported separately in other publications with a focus on the unique set of determinants arising from the “one in one hundred years pandemic” crisis.

## Conclusion

As we aspire to improve mental health services, and improve population mental health in an equitable way (86), we need population level surveillance to understand and address root causes. If inequity or other social or economic conditions are driving prevalence up, then we need models that quantify this. Perhaps these conditions are so powerful that mental health services cannot reasonably be expected alone to influence national prevalence. But services also have a part to play in the aspiration toward improving population mental health. At the very least, they should not make the situation worse. Recent interest in mental health and new funding may go some way toward bridging the gap between the level of funding and the burden of disease for which mental illness is responsible (24). In this context, resources for mental health care are precious and cannot be wasted. They should be carefully directed to where they are needed most, and to whom, including with attention to equity in service provision, then to delivery of acceptable and effective kinds of help. Effective actions must also model broader cooperation across a mental health “ecosystem” (87), and attend to social determinants of mental health in economic, housing, educational, employment and other policy spheres across government portfolios and with the community. This is perhaps Australia’s greatest challenge in mental health reform now, beyond the usual calls for political will and more funding. Informed by contemporary modeling and paying particular attention to equitable implementation of evidence-based care, treatment and recovery support, we should be seeking to set and implement a broad and bold agenda for planning and reform, one that could provide all Australians with enjoyment of the greatest attainable standard of mental health.

## Data Availability Statement

The original contributions presented in the study are included in the article/Supplementary Material. The original data can be obtained by contacting the Australian Bureau of Statistics.

## Ethics Statement

Ethical review and approval was not required for the study on human participants in accordance with the local legislation and institutional requirements. Written informed consent for participation was not required for this study in accordance with the national legislation and the institutional requirements.

## Author Contributions

JE and GM were responsible for the conception and design of the study. JE and SD were responsible for the acquisition of data and analysis. All authors made contributions to the interpretation of data, drafting the article or revising it critically for important intellectual content, and approval of the version to be submitted.

## Conflict of Interest

The authors declare that the research was conducted in the absence of any commercial or financial relationships that could be construed as a potential conflict of interest.

## Publisher’s Note

All claims expressed in this article are solely those of the authors and do not necessarily represent those of their affiliated organizations, or those of the publisher, the editors and the reviewers. Any product that may be evaluated in this article, or claim that may be made by its manufacturer, is not guaranteed or endorsed by the publisher.
