# Corrigendum: Mental Health in Australia: Psychological Distress Reported in Six Consecutive Cross-Sectional National Surveys From 2001 to 2018

**DOI:** 10.3389/fpsyt.2022.934065

**Published:** 2022-06-27

**Authors:** Joanne Enticott, Shrinkhala Dawadi, Frances Shawyer, Brett Inder, Ellie Fossey, Helena Teede, Sebastian Rosenberg, Ingrid Ozols AM, Graham Meadows

**Affiliations:** ^1^Southern Synergy, Department of Psychiatry, Monash University, Melbourne, VIC, Australia; ^2^Monash Centre for Health Research and Implementation, Monash University, Clayton, VIC, Australia; ^3^Monash Business School, Monash University, Melbourne, VIC, Australia; ^4^Department of Occupational Therapy, Monash University Peninsula Campus, Melbourne, VIC, Australia; ^5^School of Primary and Allied Health Care, Monash University, Victoria, VIC, Australia; ^6^Brain and Mind Centre, Sydney Medical School, University of Sydney, Sydney, NSW, Australia; ^7^Mental Health at Work, Melbourne, VIC, Australia; ^8^Centre for Mental Health, School of Population and Global Health, University of Melbourne, Melbourne, VIC, Australia; ^9^Monash Health, Dandenong, VIC, Australia

**Keywords:** psychological distress, mental health services, prevalence, population measures, mental health

In the original article, there was an error. Unfortunately, some important text in the discussion was misplaced.

A correction has been made to **Discussion**, “*Changes Vary Between Subgroups*,” paragraph one:

The original text, “Very-high distress in women aged 55–64 has doubled this century (from 3.5 to 7.2%) and combined high/very-high distress has increased by 50% (12.4–18.7%), both of which are highly significant findings. Very-high distress also increased in males, significantly in those aged 25–34 years. Overall, distress was greatest in women aged 18–24 years during all years; 8.0% for very-high levels and 22.1% for combined high/very-high in 2017/18. (2.1–4.0%), but overall is a more tentative finding since a significant increase did not extend to the combined high/very-high distress metric (10.6–11.5%).” has been corrected to:

“Very-high distress in women aged 55–64 has doubled this century (from 3.5 to 7.2%) and combined high/very-high distress has increased by 50% (12.4–18.7%), both of which are highly significant findings. Very-high distress also increased in males, significantly in those aged 25–34 years, but this is a more tentative finding since a significant increase did not extend to the combined high/very high distress metric (10.6–11.5%). Overall, distress was greatest in women aged 18–24 years during all years; 8.0% for very-high levels and 22.1% for combined high/very-high in 2017/18 (2.1–4.0%).”

The authors apologize for this error and state that this does not change the scientific conclusions of the article in any way. The original article has been updated.

## Publisher's Note

All claims expressed in this article are solely those of the authors and do not necessarily represent those of their affiliated organizations, or those of the publisher, the editors and the reviewers. Any product that may be evaluated in this article, or claim that may be made by its manufacturer, is not guaranteed or endorsed by the publisher.

