# Supplementary material for: Mental Health in Australia: Psychological Distress Reported in Six Consecutive Cross-Sectional National Surveys From 2001 to 2018
Source: Front Psychiatry. 2022 Apr 1;13:815904. doi: 10.3389/fpsyt.2022.815904 (PMC9010616; doi:10.3389/fpsyt.2022.815904)
Supplement: Supplementary file 1 [file Data_Sheet_1.pdf]

Supplementary Tables

Supplementary Table 1: Odds Ratio of psychological distress adjusted for year, sex, age and residential location.

		K10 very-high				K10 combined high/very-high			
		Odds Ratio	p-value	[95% Conf.	Interval]	Odds Ratio	p-value	[95% Conf.	Interval]
**Year**	2001	(Ref)	-	-		(Ref)	-	-	
	2004	1.03	0.602	0.92	1.15	1.01	0.705	0.95	1.08
	2007	0.92	0.168	0.81	1.04	0.92	0.017*	0.86	0.98
	2011	0.95	0.369	0.84	1.07	0.81	<0.001 ***	0.76	0.88
	2014	1.07	0.250	0.95	1.21	0.91	0.011*	0.85	0.98
	2017	1.24	<0.001 ***	1.11	1.39	1.08	0.033*	1.01	1.15
**Sex**	Males	(Ref)	-	-		(Ref)	-	-	
	Females	1.56	<0.001 ***	1.45	1.67	1.54	<0.001 ***	1.47	1.60
**Age (years)**	18 - 24	(Ref)	-	-		(Ref)	-	-	
	25 - 34	0.86	0.032*	0.75	0.99	0.77	<0.001 ***	0.72	0.83
	35 - 44	0.97	0.646	0.85	1.11	0.79	<0.001 ***	0.73	0.85
	45 - 54	1.33	<0.001 ***	1.17	1.52	0.86	<0.001 ***	0.80	0.93
	55 - 64	1.10	0.168	0.96	1.26	0.79	<0.001 ***	0.73	0.85
**Location**	Major cities	(Ref)	-	-		++++++ -	-	-	
	Inner regional	1.16	0.001**	1.06	1.26	1.11	<0.001 ***	1.05	1.17
	Other	1.03	0.505	0.94	1.14	1.06	0.069	1.00	1.12

Supplementary Table 2: Prevalence of psychological distress by sex and age-groups over time.

	K10 very-high				K10 combined high/very-high		
	n	Rate	95%	CI	Rate	95%	CI
Males aged 18-24 years							
2001	759	2.64%	1.50%	3.77%	11.07%	8.84%	13.30%
2004	622	2.25%	1.09%	3.42%	11.25%	8.77%	13.74%
2007	684	1.02%	0.27%	1.78%	7.31%	5.36%	9.26%
2011	626	2.72%	1.44%	3.99%	10.86%	8.43%	13.30%
2014	560	3.93%	2.32%	5.54%	11.61%	8.95%	14.26%
2017	562	3.91%	2.31%	5.52%	15.30%	12.33%	18.28%
Males aged 25-34 years							
2001	1555	2.12%	1.41%	2.84%	10.61%	9.08%	12.14%
2004	1569	2.55%	1.77%	3.33%	9.82%	8.34%	11.29%
2007	1224	2.29%	1.45%	3.13%	10.54%	8.82%	12.26%
2011	1231	2.11%	1.31%	2.92%	9.10%	7.49%	10.70%
2014	1106	2.98%	1.98%	3.99%	9.40%	7.68%	11.12%
2017	1205	3.98%	2.88%	5.09%	11.45%	9.65%	13.25%
Males aged 35-44 years							
2001	1888	2.81%	2.06%	3.55%	10.43%	9.06%	11.81%
2004	1865	3.65%	2.80%	4.50%	12.06%	10.59%	13.54%
2007	1553	2.96%	2.12%	3.81%	9.98%	8.49%	11.47%
2011	1427	3.15%	2.25%	4.06%	9.46%	7.94%	10.98%
2014	1253	3.19%	2.22%	4.17%	10.69%	8.98%	12.41%
2017	1307	3.21%	2.26%	4.17%	10.64%	8.96%	12.31%
Males aged 45-54 years							
2001	1544	4.02%	3.04%	4.99%	11.53%	9.94%	13.12%
2004	1670	3.95%	3.02%	4.89%	12.16%	10.59%	13.72%
2007	1395	4.16%	3.11%	5.21%	11.47%	9.80%	13.14%
2011	1353	3.55%	2.56%	4.53%	9.16%	7.63%	10.70%
2014	1164	3.95%	2.83%	5.07%	10.91%	9.12%	12.70%
2017	1330	5.11%	3.93%	6.30%	13.08%	11.27%	14.90%
Males aged 55-64 years							
2001	1051	3.62%	2.49%	4.74%	10.75%	8.88%	12.62%
2004	1409	3.83%	2.83%	4.83%	11.36%	9.70%	13.01%
2007	1239	3.63%	2.59%	4.67%	11.70%	9.91%	13.49%
2011	1234	4.13%	3.02%	5.24%	9.56%	7.92%	11.20%
2014	1114	3.23%	2.19%	4.27%	9.16%	7.46%	10.85%
2017	1310	5.04%	3.85%	6.22%	13.89%	12.02%	15.77%
Females aged 18-24 years							
2001	869	5.64%	4.11%	7.17%	23.02%	20.22%	25.81%
2004	708	3.25%	1.94%	4.55%	19.63%	16.71%	22.56%
2007	679	4.71%	3.12%	6.31%	18.41%	15.49%	21.32%
2011	600	4.00%	2.43%	5.57%	15.50%	12.60%	18.40%
2014	565	6.90%	4.81%	8.99%	21.42%	18.03%	24.80%
2017	612	8.01%	5.86%	10.16%	22.06%	18.77%	25.34%
Females aged 25-34 years							
2001	1924	5.35%	4.35%	6.36%	17.15%	15.47%	18.84%
2004	1802	4.50%	3.54%	5.45%	16.04%	14.34%	17.73%
2007	1442	3.88%	2.89%	4.88%	14.36%	12.55%	16.16%
2011	1483	3.51%	2.57%	4.44%	13.01%	11.30%	14.73%
2014	1380	3.91%	2.89%	4.94%	13.12%	11.33%	14.90%

2017	1371	4.30%	3.23%	5.38%	15.17%	13.27%	17.07%
Females aged 35-44 years							
2001	2172	4.65%	3.76%	5.54%	16.44%	14.88%	18.00%
2004	2148	5.49%	4.53%	6.46%	17.32%	15.72%	18.92%
2007	1666	4.44%	3.45%	5.43%	14.83%	13.12%	16.53%
2011	1548	4.07%	3.09%	5.05%	12.73%	11.07%	14.39%
2014	1510	4.57%	3.52%	5.62%	13.64%	11.91%	15.37%
2017	1537	4.49%	3.45%	5.52%	14.12%	12.38%	15.86%
Females aged 45-54 years							
2001	1664	6.43%	5.25%	7.61%	16.53%	14.74%	18.31%
2004	1839	6.58%	5.45%	7.71%	16.53%	14.83%	18.23%
2007	1451	5.58%	4.40%	6.76%	15.78%	13.91%	17.66%
2011	1507	7.03%	5.74%	8.32%	14.86%	13.07%	16.66%
2014	1355	6.64%	5.32%	7.97%	16.75%	14.76%	18.74%
2017	1470	6.94%	5.64%	8.24%	17.48%	15.54%	19.42%
Females aged 55-64 years							
2001	1215	3.54%	2.50%	4.58%	12.35%	10.50%	14.20%
2004	1536	4.56%	3.51%	5.60%	14.26%	12.51%	16.01%
2007	1271	4.41%	3.28%	5.53%	14.00%	12.10%	15.91%
2011	1323	4.31%	3.21%	5.40%	13.00%	11.19%	14.81%
2014	1289	6.05%	4.75%	7.35%	14.58%	12.66%	16.51%
2017	1479	7.17%	5.85%	8.48%	18.73%	16.74%	20.72%

Supplementary Table 3: Prevalence of psychological distress by household income over time. Income quintile 1 are lowest incomes, and quintile 5 are highest.

		**K10 very-high**			**K10 combined high/very-high**		
2001	n	Mean	[95%	CI]	Mean	[95%	CI]
1	2064	10.88%	9.25%	12.51%	24.56%	22.36%	26.76%
2	1845	6.06%	4.78%	7.35%	20.11%	17.95%	22.26%
3	2201	1.98%	1.41%	2.56%	11.82%	10.27%	13.37%
4	2576	2.14%	1.47%	2.81%	9.12%	7.82%	10.43%
5	3077	1.01%	0.58%	1.45%	5.76%	4.82%	6.70%
2004							
1	2286	11.47%	9.90%	13.05%	28.36%	26.07%	30.65%
2	2071	5.39%	4.19%	6.58%	16.99%	15.00%	18.99%
3	2547	3.33%	2.45%	4.20%	12.87%	11.23%	14.50%
4	2909	1.82%	1.24%	2.39%	10.09%	8.75%	11.43%
5	3257	1.07%	0.64%	1.50%	5.69%	4.73%	6.66%
2007							
1	1518	10.12%	8.26%	11.97%	26.02%	23.11%	28.94%
2	1824	5.13%	3.86%	6.39%	16.69%	14.50%	18.88%
3	2340	3.22%	2.04%	4.40%	11.18%	9.39%	12.97%
4	2656	2.23%	1.43%	3.03%	9.68%	8.19%	11.17%
5	2823	1.13%	0.58%	1.68%	6.53%	5.31%	7.75%
2011							
1	1286	10.80%	8.69%	12.92%	25.81%	22.70%	28.92%
2	1477	6.74%	5.18%	8.30%	17.44%	15.06%	19.82%
3	2175	3.07%	2.22%	3.92%	10.03%	8.48%	11.59%
4	2595	1.68%	0.99%	2.37%	7.07%	5.77%	8.36%
5	2730	1.43%	0.83%	2.04%	5.59%	4.48%	6.70%
2014							
1	1342	11.77%	9.67%	13.86%	25.85%	22.76%	28.95%
2	1297	6.52%	4.77%	8.26%	19.37%	16.59%	22.15%
3	1880	3.02%	2.04%	4.00%	12.30%	10.37%	14.22%
4	2289	1.99%	1.21%	2.78%	9.13%	7.53%	10.73%
5	2346	1.73%	0.90%	2.57%	5.65%	4.39%	6.92%
2017							
1	1539	10.72%	8.99%	12.45%	26.42%	23.68%	29.15%
2	1541	6.66%	5.12%	8.20%	18.97%	16.50%	21.44%
3	2164	3.61%	2.61%	4.61%	13.00%	11.20%	14.80%
4	2576	1.89%	1.23%	2.55%	9.19%	7.80%	10.58%
5	2664	2.11%	1.30%	2.91%	7.98%	6.61%	9.36%

Supplementary Table 4: Prevalence of psychological distress by household income quintile and time. Income quintile 1 are lowest incomes, and quintile 5 are highest.

	K10 very-high				K10 combined high/very-high		
	**n**	**Rate**	**95%**	**CI**	**Rate**	**95%**	**CI**
Males income quintile 1 (poorest)							
2001	820	9.64%	7.13%	12.16%	21.71%	18.34%	25.08%
2004	877	11.51%	8.88%	14.14%	30.14%	26.34%	33.94%
2007	669	8.93%	6.21%	11.65%	24.90%	20.48%	29.32%
2011	605	10.52%	7.43%	13.60%	26.17%	21.61%	30.73%
2014	530	9.33%	6.31%	12.35%	22.67%	17.99%	27.36%
2017	619	9.98%	7.43%	12.53%	24.64%	20.35%	28.93%
Males income quintile 2							
2001	689	5.12%	3.21%	7.03%	15.99%	12.86%	19.11%
2004	836	5.66%	3.70%	7.62%	14.16%	11.29%	17.02%
2007	779	4.68%	2.65%	6.71%	14.53%	11.39%	17.66%
2011	610	5.56%	3.39%	7.74%	14.32%	10.98%	17.66%
2014	498	5.20%	2.66%	7.75%	17.06%	12.76%	21.36%
2017	638	4.68%	2.97%	6.39%	13.41%	10.50%	16.32%
Males income quintile 3							
2001	1054	1.45%	0.77%	2.13%	8.70%	6.82%	10.58%
2004	1201	2.67%	1.65%	3.70%	10.75%	8.58%	12.93%
2007	1106	1.82%	0.78%	2.85%	8.06%	6.00%	10.12%
2011	1001	2.39%	1.21%	3.56%	8.08%	6.03%	10.12%
2014	864	2.19%	1.01%	3.37%	10.03%	7.49%	12.57%
2017	1003	3.18%	1.85%	4.50%	11.98%	9.49%	14.47%
Males income quintile 4							
2001	1234	1.54%	0.75%	2.33%	6.88%	5.29%	8.47%
2004	1511	0.96%	0.37%	1.55%	7.14%	5.55%	8.74%
2007	1324	1.92%	0.79%	3.06%	7.61%	5.72%	9.50%
2011	1276	0.95%	0.32%	1.57%	5.27%	3.75%	6.80%
2014	1143	1.63%	0.70%	2.57%	7.62%	5.49%	9.75%
2017	1273	1.82%	0.98%	2.67%	9.08%	7.17%	11.00%
Males income quintile 5 (richest)							
2001	1703	0.43%	0.01%	0.85%	4.16%	3.09%	5.24%
2004	1785	0.60%	0.24%	0.96%	4.02%	2.96%	5.08%
2007	1564	0.91%	0.30%	1.52%	5.38%	3.90%	6.86%
2011	1476	0.94%	0.33%	1.54%	4.25%	2.95%	5.56%
2014	1222	1.48%	0.24%	2.73%	4.22%	2.56%	5.87%
2017	1411	1.68%	0.61%	2.76%	6.28%	4.49%	8.06%
Females income quintile 1 (poorest)							
2001	1244	11.91%	9.78%	14.04%	26.94%	24.05%	29.82%
2004	1409	11.45%	9.50%	13.39%	27.10%	24.26%	29.93%
2007	849	11.14%	8.59%	13.68%	26.99%	23.11%	30.88%
2011	681	11.08%	8.18%	13.98%	25.46%	21.23%	29.69%
2014	812	13.63%	10.74%	16.52%	28.29%	24.18%	32.40%
2017	920	11.30%	8.95%	13.65%	27.82%	24.29%	31.35%
Females income quintile 2							
2001	1156	6.80%	5.07%	8.53%	23.31%	20.37%	26.24%
2004	1235	5.15%	3.71%	6.59%	19.43%	16.68%	22.18%
2007	1045	5.50%	3.91%	7.09%	18.49%	15.45%	21.53%
2011	867	7.74%	5.54%	9.95%	20.09%	16.74%	23.43%

2014	799	7.60%	5.21%	9.98%	21.27%	17.65%	24.90%
2017	903	8.21%	5.84%	10.58%	23.32%	19.65%	27.00%
Females income quintile 3							
2001	1147	2.53%	1.59%	3.46%	14.99%	12.55%	17.43%
2004	1346	3.95%	2.55%	5.35%	14.88%	12.47%	17.29%
2007	1234	4.47%	2.46%	6.49%	13.98%	11.16%	16.80%
2011	1174	3.68%	2.46%	4.89%	11.77%	9.47%	14.08%
2014	1016	3.86%	2.30%	5.41%	14.59%	11.71%	17.47%
2017	1161	4.03%	2.53%	5.53%	13.99%	11.40%	16.58%
Females income quintile 4							
2001	1342	2.75%	1.66%	3.84%	11.44%	9.38%	13.49%
2004	1398	2.82%	1.78%	3.86%	13.53%	11.31%	15.75%
2007	1332	2.55%	1.41%	3.69%	11.86%	9.55%	14.17%
2011	1319	2.43%	1.20%	3.66%	8.90%	6.81%	10.99%
2014	1146	2.37%	1.10%	3.65%	10.73%	8.33%	13.13%
2017	1303	1.97%	0.95%	2.99%	9.31%	7.29%	11.32%
Females income quintile 5 (richest)							
2001	1374	1.75%	0.92%	2.58%	7.79%	6.16%	9.42%
2004	1472	1.67%	0.81%	2.52%	7.83%	6.13%	9.54%
2007	1259	1.42%	0.43%	2.40%	8.02%	6.00%	10.05%
2011	1254	2.08%	0.92%	3.23%	7.31%	5.43%	9.19%
2014	1124	2.03%	0.97%	3.09%	7.36%	5.40%	9.32%
2017	1253	2.62%	1.41%	3.84%	10.07%	7.93%	12.20%
